# The impact of social media presence on primary care sports medicine fellowship recruitment: a cross-sectional study

**DOI:** 10.1186/s12909-025-07640-7

**Published:** 2025-07-22

**Authors:** Tiana S. Woolridge, Cooper Bloyd, Madelynn Taylor, Nicolas Hatamiya, Celina de Borja

**Affiliations:** 1 David Geffen School of Medicine at UCLA, Department of Family Medicine, Sports Medicine Fellowship Program, 1920 Colorado Avenue, Santa Monica, CA 90404 USA; 2 Department of Pediatrics, University of California, San Francisco, Campus Box 0110, 550 16th Street, San Francisco, CA 94143 USA; 3 Departments of Orthopaedic Surgery and Family & Community Medicine, University of California, San Francisco, 1500 Owens Street, #200, San Francisco, CA 94158 USA; 4 Departments of Orthopaedic Surgery and Pediatrics, University of California, San Francisco, 1825 4th St, 5B, San Francisco, CA 94158 USA

**Keywords:** Graduate Medical Education, Social media, Recruitment, Fellowship, Primary Care Sports Medicine

## Abstract

**Background:**

The COVID-19 pandemic limited in-person recruitment opportunities for many training programs at academic medical centers. This study aimed to determine the prevalence of social media use among Primary Care Sports Medicine (PCSM) fellowship programs and to investigate the impact of social media on recruitment. Additionally, potential barriers to social media interaction between programs and applicants were examined.

**Methods:**

A cross-sectional study was conducted using publicly available online sources to identify PCSM fellowship programs and corresponding social media accounts. PCSM Fellowship directors were surveyed regarding their program's use of social media. Prospective PCSM Fellowship Applicants and current fellows were surveyed to determine their interaction with PCSM fellowship social media accounts and how social media influenced their perception of the programs.

**Results:**

A total of 211 PCSM fellowship programs were identified on the American Medical Society for Sports Medicine (AMSSM) website, of which 83% had a social media presence. The most commonly used platforms were Instagram (43%), Facebook (30%) and Twitter (24%). Fifty-eight individuals who were either current fellows, recently graduated fellows, or current fellowship applicants responded to our survey. Among the surveyed fellows and applicants, 95% reported using social media, with 35% reported following PCSM accounts. Of those who did not follow (*N* = 38, 65%), the most common reason was lack of awareness of these accounts. Twenty-two percent of respondents indicated that social media positively influenced their perception of a program and its rank list position, while the remainder reported a neutral effect. Forty-seven percent of the fellowship directors reported their program did not have official guidelines for appropriate social media use. Lack of time, resources, and oversight were identified as the most common barriers to social media use.

**Conclusions:**

A majority of PCSM fellowship programs, fellows, and applicants are present on social media. Social media presence can positively impact an applicant’s view of a program. However, applicants’ lack of awareness of these accounts may serve as a barrier for interaction. The development of universal guidelines for appropriate social media use may help increase adoption and utilization of social media platforms for PCSM fellowship recruitment, particularly during times when in-person recruitment opportunities are limited.

**Supplementary Information:**

The online version contains supplementary material available at 10.1186/s12909-025-07640-7.

## Background

The COVID-19 pandemic significantly affected the recruitment process of many academic medical centers' training programs. In-person recruitment opportunities including campus tours and on-site interviews were limited due to federal and hospital regulations, driving program leadership to develop novel strategies to engage with prospective applicants. This includes the use of social media as a means of promoting their training programs and attracting qualified applicants) [1–6]. Prior research has shown that these programs increased their social media presence in response to the COVID-19 pandemic [7]; for example, a study of orthopedic surgery program directors (PD) showed that 79% of respondents reported generating increased social media content in response to COVID-19 restrictions [8].

Social media use by academic training programs has expanded over the last decade for a variety of purposes, including medical education, engagement with the general public, and disseminating program and trainee accomplishments [2, 9, 10]. Many individuals in this generation of applicants, having grown up in the digital era, are accustomed to using social media as a means to discover information, and may be more apt than prior generations at utilizing social media when seeking knowledge about a medical training program [9].

The positive impact of social media on the perception and intended rank position of programs has been previously established for applicants seeking positions in medical residency programs, with cited benefits including helping the program highlight its culture, camaraderie among trainees, faculty, and staff, and other intangible aspects of the program [10–18]. However, the prevalence and impact of social media use in fellowship programs, specifically Primary Care Sports Medicine (PCSM) fellowship programs, have not been published. In this study, we aimed to determine the prevalence of social media use in PCSM fellowship programs, its impact on recruitment, and potential barriers to social media interaction between programs and applicants.

## Methods

A complete list of primary care sports medicine fellowship programs was collected from the American Medical Society of Sports Medicine (AMSSM) website. Information on the prevalence of social media accounts created and managed by primary care sports medicine fellowship programs was gathered in September 2022 using publicly available online sources including Google search engine as well as social media website search functions on Instagram, Twitter, LinkedIn, Facebook, and YouTube.

This study also utilized anonymous survey instruments with a cross-sectional approach to query PCSM fellowship directors, PCSM fellows and recent fellowship graduates, and PCSM program applicants regarding their interaction with PCSM fellowship social media accounts and how social media influenced the perception of the programs during their application process. The first survey was disseminated through individual outreach to all fellowship directors of each primary care sports medicine fellowship program in the United States of America, as identified through AMSSM, which sought to identify PDs’’ current use of and comfort level with utilizing social media as a recruitment tool for their program. A second survey with the intention to garner applicant perspectives on social media for recruitment was disseminated to current fellows through individual outreach to all program coordinators of each primary care sports medicine fellowship program, who were then asked to send the survey to the fellows in their program. This survey was also disseminated to recently graduated fellows and current fellowship applicants by posting a request to complete the survey on AMSSM social media pages and research platforms. The second survey queried trainee’s interaction with PCSM fellowship social media accounts, and how social media influenced the perception of the programs during the application process. Surveys were distributed from September to November 2022. Approval for this study was obtained through the University of California, San Francisco Institutional Review Board. Informed consent to participate was obtained from all of the participants in the study.

## Results

### Social media use by PCSM fellowship programs

We identified 211 PCSM fellowship programs listed on the AMSSM website. Of these 211 programs, 175 had a social media presence. For the purposes of this study, social media presence is defined as having social media content specific to the fellowship program that is accessible on publicly available social media platforms. 107 of the PCSM fellowship programs included in this study were highlighted in institutional or departmental social media accounts that were not directly run by faculty, staff, or fellows within the fellowship. Sixty-sevenprograms had at least one social media account that was exclusively run by and dedicated to the PCSM fellowship program, with the most used social media platforms being Instagram (*N* = 29, 43%), Facebook (*N* = 20, 30%), and Twitter (*N* = 16, 24%).

### Prospective applicant, current or recent fellow survey results

**Fig. 1:**
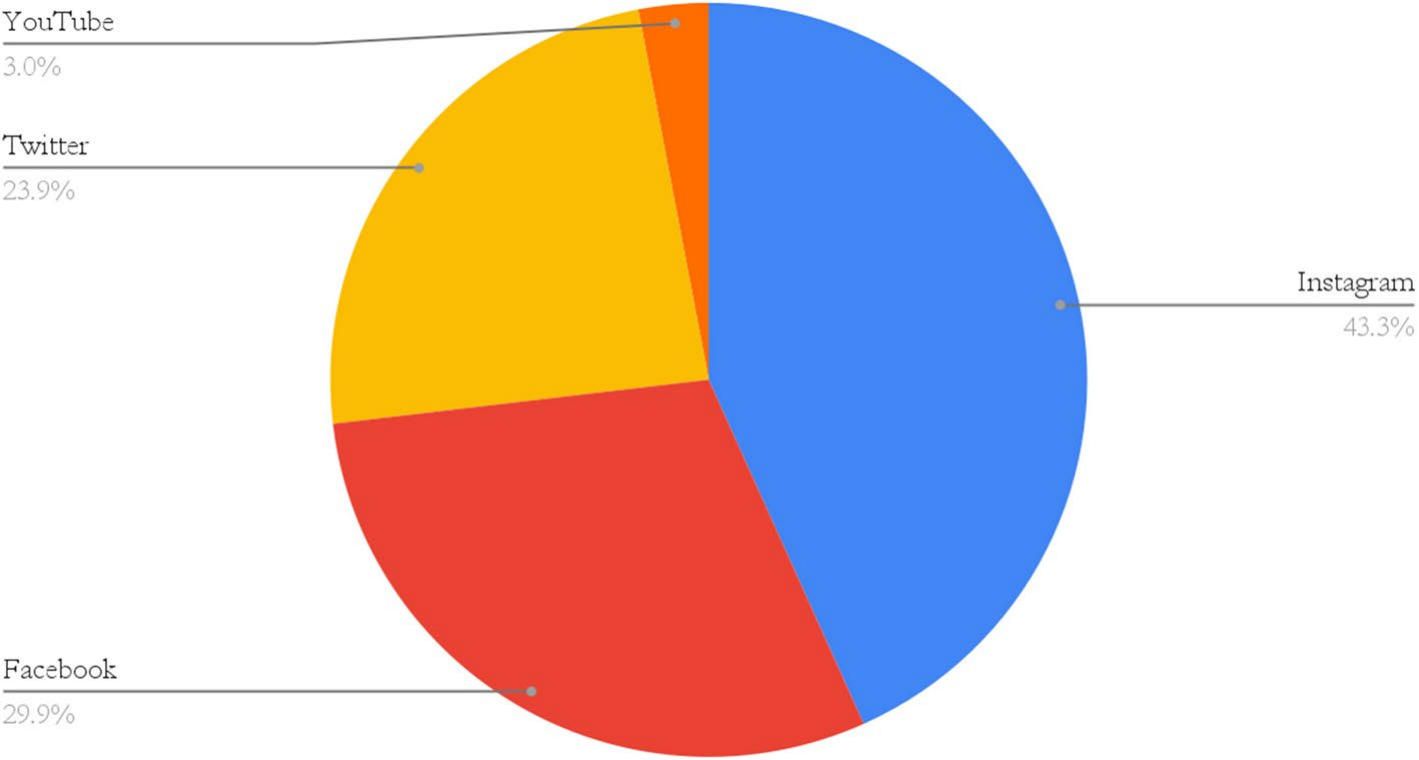
Types of social media accounts used by PCSM fellowship programs (*N* = 67)

**Fig. 2:**
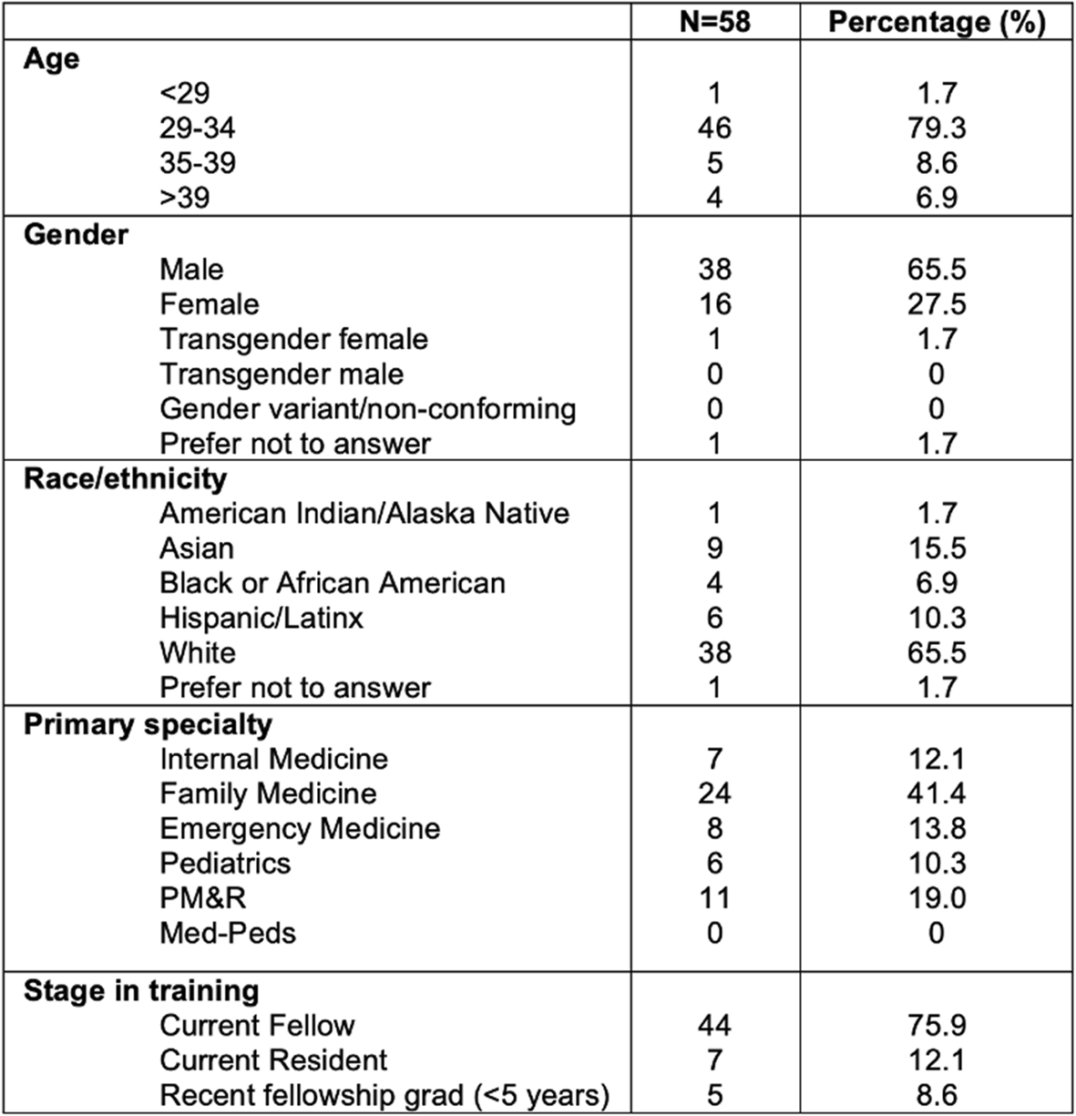
Trainee demographic information: PM&R = physical medicine and rehabilitation; Med-Peds = Medicine-Pediatrics)

Fifty-eight individuals who identified as a current fellowship applicant, current fellow, or recent fellowship graduate, defined as being less than five years out of fellowship, completed the survey from a variety of primary specialties and socioeconomic backgrounds (see Fig. 1). Of those, 95% reported using social media. Approximately half of respondents reported utilizing social media for professional use and education, in addition to personal use. While most respondents were connected to friends and family on social media, 29% reported connecting with professional organizations on social media and 25% reported connecting with medical organizations (Fig. 2).

**Fig. 3:**
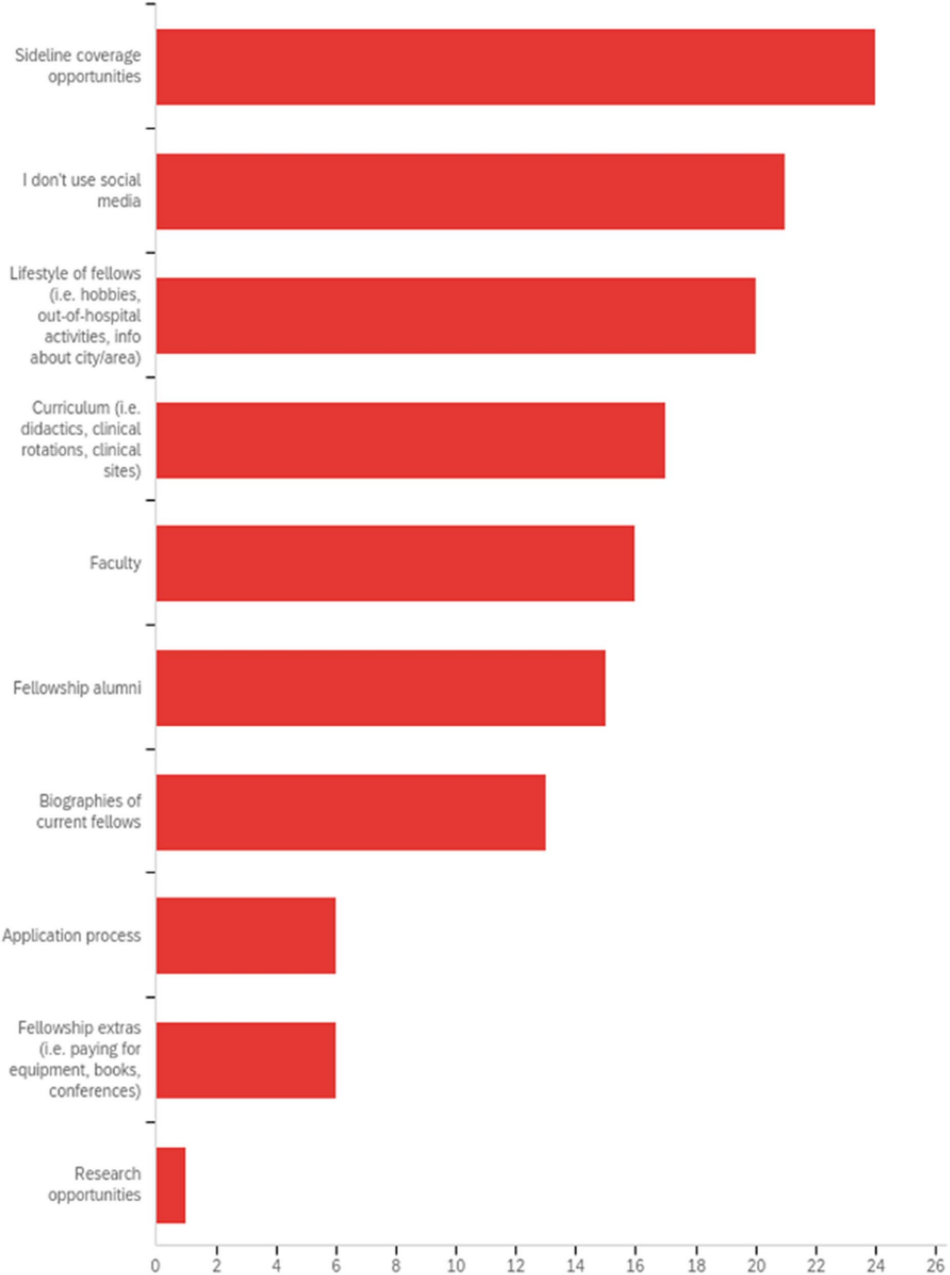
Survey responses to the following question: “If you used social media to obtain information, what information were you hoping to find on the fellowship social media accounts? Check all that apply.”

A total of 52 survey respondents (90%) reported using the fellowship website when researching information about fellowship programs during their fellowship application process, while 19% used social media to research programs. When asked what information trainees wanted to see on social media, the most reported responses included sideline coverage opportunities (*n* = 23, 40%), fellow lifestyle (hobbies, out-of-hospital activities, and information about the city in which the fellowship program is located) (*n* = 19, 33%), and fellowship curriculum (didactics, clinical rotations, and clinical sites) (*n* = 16, 28%) (Fig. 3).

In total, 35% of respondents reported following PCSM accounts. Lack of awareness of these accounts was the most common reason for not following them. Of the respondents who followed PCSM accounts, 22% reported that social media positively influenced their perception of a program and its rank list position.

### PCSM fellowship program director results

Seventy fellowship directors responded to the survey intended to gather information on PD’s current use of and comfort level with utilizing social media as a recruitment tool for their program (response rate 70/211, 33.2%). Forty-seven percent of respondents reported that their program did not have official guidelines for appropriate social media use. The most reported barriers for social media use included lack of time (32%), resources (13%), and oversight (11%).

## Discussion

Our study found that the majority of PCSM fellowship programs, fellows, and applicants are present on social media. The prevalence of social media use in PCSM fellowship programs is high, with 83% of programs having a social media presence. However, only 35% of respondents (current, past and prospective PCSM fellows) reported following PCSM accounts, indicating a lack of awareness of these accounts among prospective applicants.

Despite the limited use of PCSM social media accounts, our study found that social media presence can positively influence applicants' perception of a program and its rank list position. This finding aligns with prior research on the positive impact of social media presence on applicant perception of a program [3, 8, 12]. For example, a study conducted on first-year residents demonstrated that the majority of the respondents agreed or strongly agreed that their perception of a program was positively influenced by the residency program’s social media account [6]. Our study demonstrated that fellowship applicants would like to see content related to sports coverage experiences and fellow lifestyle, which may be better suited for social media platforms that allow for more in-depth photo and video content compared to traditional fellowship websites. Programs can use social media to showcase their culture, academic offerings, and social gatherings in a way that is easily accessible, particularly to applicants who, due to physical distance or work duties, would not otherwise be able to engage with the program through in-person interviews or clinical rotations.

Our study also identified potential barriers to social media use in PCSM fellowship programs, including lack of time, resources, and oversight. This finding aligns with prior research which also demonstrated barriers such as insufficient protected time, insufficient IT support to host a platform, and a lack of knowledge among faculty of how to utilize social media [19]. Nearly half of the fellowship directors reported not having official guidelines for appropriate social media use, which could contribute to the lack of engagement with social media platforms.

However, there is increasing interest in the use of social media for recruitment purposes; a survey of radiology showed that 38% of 132 associate PDs report social media use and roughly a quarter felt that program Facebook pages would be of value [20, 21]. Future research and the development of social media guidelines can potentially facilitate social media growth by programs by improving PDs’ level of comfort with these platforms [22]. By following guidelines such as those proposed below, graduate medical education programs can leverage social media platforms effectively for trainee recruitment while upholding principles of patient privacy, professionalism, and diversity, equity, and inclusion Table 1.

**Table 1:** Sample guidelines for appropriate social media use by graduate medical education programs

*Proposed Guidelines for Social Media Use for Trainee Recruitment by Graduate Medical Education Programs*
1. Prioritize Patient Privacy: Ensure that all social media content complies with patient privacy regulations. Avoid sharing identifiable patient information or images without explicit consent
2. Maintain Professionalism: Trainees, faculty, and staff representing the program should adhere to professional codes of conduct and avoid engaging in unprofessional behavior online such as sharing confidential patient information, engaging in derogatory language or discriminatory remarks, or posting inappropriate content that could reflect negatively on themselves or the program
3. Educate Trainees on Social Media Etiquette: Provide education and training to trainees on appropriate social media use and etiquette. Help them understand the potential impact of their online presence on their professional reputation and the reputation of the program
4. Use Inclusive Language: Use language that is inclusive and respectful of all individuals, regardless of race, ethnicity, gender identity, sexual orientation, disability, or other characteristics. Avoid language that may perpetuate stereotypes or biases
5. Promote Diversity, Equity, and Inclusion (DEI): Actively promote diversity, equity, and inclusion in social media content and recruitment efforts. Showcase the program's commitment to diversity by highlighting diverse faculty, trainees, and patients, and the ways in which the program supports underrepresented populations
6. Monitor and Moderate Content: Regularly monitor social media channels associated with the program to ensure that content aligns with program values and objectives
7. Engage Responsibly with Prospective Trainees: Engage with prospective trainees on social media platforms in a respectful and professional manner. Provide accurate information about the program and respond promptly to inquiries or feedback
8. Evaluate and Adapt Strategies: Continuously evaluate the effectiveness of social media recruitment strategies in attracting diverse and qualified candidates. Solicit feedback from trainees, faculty, and staff to identify areas for improvement and adapt strategies accordingly

Several limitations should be taken into account when interpreting the results of this study. The convenience sampling methods used to recruit participants may have biased responses towards those who are more likely to use social media or rely on electronic resources to guide decision-making. Therefore, the findings may not be generalizable to all PCSM fellowship applicants and programs. Additionally, the limited survey response rate from PDs may have introduced nonresponse bias and affected the representativeness of the sample. The response rate of fellows and applicants was unable to be calculated given the convenience sampling nature of the study; the survey was distributed via listservs, posts on several social media channels, and word-of-mouth. However, reassuringly, the study demographics closely matched that of the American Medical Society of Sports Medicine population of primary care sports medicine physicians for gender and race/ethnicity.

## Conclusions

There has been increased attention on virtual recruitment of graduate medical education programs and social media has been shown to positively impact applicants’ perception of programs. Our study is the first to evaluate the impact of social medical use in the recruitment of PCSM fellows. A majority of PCSM fellowship programs had a social media presence, with Instagram, Facebook, and Twitter being the most commonly used platforms, respectively. Social media presence may positively influence the perception of a program, while lack of awareness may serve as a barrier for interaction. The development of universal guidelines for appropriate social media use may help increase utilization of social media platforms for PCSM fellowship recruitment, particularly during times when in-person recruitment opportunities are limited.

## Supplementary Information

Supplementary Material 1.

## Data Availability

Data collected on social media accounts of primary care sports medicine programs can be found here: https://docs.google.com/spreadsheets/d/1LcAh-TZ5TwCdAzgWzUyxXa0IIom1l70WDjiernpMEtw/edit?usp=drive_link. Data on the survey responses and demographic information is provided within the manuscript.

## Abbreviation

PCSM: Primary care sports medicine
