# Supplementary material for: The impact of social media presence on primary care sports medicine fellowship recruitment: a cross-sectional study
Source: BMC Med Educ. 2025 Jul 22;25:1094. doi: 10.1186/s12909-025-07640-7 (PMC12282002; doi:10.1186/s12909-025-07640-7)
Supplement: Supplementary file 1 — Supplementary Material 1. [file 12909_2025_7640_MOESM1_ESM.docx]

**Appendix**

**Social Media in PCSM Fellowship Programs Survey**

*Note: social media is defined as* [*forms*](https://dictionary.cambridge.org/us/dictionary/english/form) *of* [*media*](https://dictionary.cambridge.org/us/dictionary/english/media) *that* [*allow*](https://dictionary.cambridge.org/us/dictionary/english/allow) [*people*](https://dictionary.cambridge.org/us/dictionary/english/people) *to* [*communicate*](https://dictionary.cambridge.org/us/dictionary/english/communicate) *and* [*share*](https://dictionary.cambridge.org/us/dictionary/english/share) [*information*](https://dictionary.cambridge.org/us/dictionary/english/information) *using the* [*internet*](https://dictionary.cambridge.org/us/dictionary/english/internet) *or* [*mobile*](https://dictionary.cambridge.org/us/dictionary/english/mobile) [*phones*](https://dictionary.cambridge.org/us/dictionary/english/phone)*. For the purposes of this study, a program website is not considered to fall under the category of social media.*

1. Does your fellowship program have an official Social Media outlet?

☐ Yes ☐ No

2. What form(s) of Social Media does your fellowship program utilize? (Check all that apply).

☐ Facebook

☐ Twitter

☐ LinkedIn

☐ Instagram

☐ YouTube

☐ Blogs

☐ Podcasts

☐ Other (fill in the blank) ________________

3. How comfortable are you with having a social media presence for your program?

☐ Very comfortable

☐ Relatively comfortable

☐ Neutral

☐ Relatively uncomfortable

☐ Not very comfortable

4. Do you have an official policy or guideline that outlines appropriate Social Media use by your fellowship program?

☐ Yes ☐ No

5. In your opinion, what is the most important content to post on social media in order to recruit potential applicants?

Please rank, with 1 being the most important:

a. Fellow life (out of office/hospital activities)

b. Fellow/faculty/alumni accomplishments (awards, recognitions)

c. Fellow educational opportunities (didactics, rotations, coverage)

d. Research (published articles, grants)

e. Other

6. How often is new content created or posted on Social Media by your fellowship program? (Approximately)

☐ None

☐ Once per month

☐ Once per week

☐ Once per day

☐ Other (please specify)

9. What do you feel are barriers to Social Media use in your fellowship program?

- - (Check all that apply)

☐ Do not have time to use or implement

☐ Lack of familiarity with social media

☐ Lack of resources for social media

☐ Too distracting or disruptive

☐ Lack of participation

☐ Lack of professionalism

☐ Lack of privacy

☐ Lack of oversight on the quality of content posted

☐ Legal concerns

☐ None

☐ Other (please specify)

15. Does your fellowship program have dedicated funding and/or resources to support Social Media use?

☐ Yes ☐ No

16. Does your program have a dedicated staff member (not a current fellow) to maintain and update your program's Social Media accounts?

☐ Yes ☐ No

17. How valuable do you perceive the use of social media for your fellowship program?

☐ Very valuable

☐ Somewhat valuable

☐ Unsure

☐ Somewhat not valuable

☐ No value

**The Impact of Social Media on Primary Care Sports Medicine (PCSM) Fellowship Applicants**

*Note: social media is defined as* [*forms*](https://dictionary.cambridge.org/us/dictionary/english/form) *of* [*media*](https://dictionary.cambridge.org/us/dictionary/english/media) *that* [*allow*](https://dictionary.cambridge.org/us/dictionary/english/allow) [*people*](https://dictionary.cambridge.org/us/dictionary/english/people) *to* [*communicate*](https://dictionary.cambridge.org/us/dictionary/english/communicate) *and* [*share*](https://dictionary.cambridge.org/us/dictionary/english/share) [*information*](https://dictionary.cambridge.org/us/dictionary/english/information) *using the* [*internet*](https://dictionary.cambridge.org/us/dictionary/english/internet) *or* [*mobile*](https://dictionary.cambridge.org/us/dictionary/english/mobile) [*phones*](https://dictionary.cambridge.org/us/dictionary/english/phone)*. For the purposes of this study, a program website is not considered to fall under the category of social media.*

**Demographics**

1. Please select your age.

a. <29

b. 29-34

c. 35-39

d. >40

2. Please select your gender.

a. Female

b. Male

c. Transgender female

d. Transgender male

e. Gender Variant/Non-Conforming

f. Prefer Not to Answer

3. Please select your race/ethnicity (check all that apply).

a. American Indian or Alaska Native

b. Asian

c. Black or African American

d. Hispanic or Latinx

e. Native Hawaiian or other Pacific Islander

f. White

g. Prefer Not to Answer

4. Please select your primary specialty.

a. Internal Medicine

b. Family Medicine

c. Emergency Medicine

d. Pediatrics

e. Physical Medicine & Rehabilitation

F. Med/Peds

5. Please select your current stage in training.

a. Resident

b. Fellow

**Social Media Use**

1. Do you use any of the following social media outlets?

a. Facebook

b. Instagram

c. Twitter

d. LinkedIn

e. Snapchat

f. Other

g. None

Please provide explanation of your selection of “other” in the previous question (optional) _________

2. For which reasons do you normally use these outlets? Check all that apply:

a. Personal use

b. Professional use

c. Education

d. News source

e. Other

Please provide explanation of your selection of “other” in the previous question (optional) _________

3. With whom are you “connected” to on your social media outlets? Check all that apply.

a. Friends

b. Family

c. Co-workers/colleagues

d. Patients

e. Professional organizations

f. Medical organizations

g. News/media

h. Other

Please provide explanation of your selection of “other” in the previous question (optional) _________

**4.** How often do you typically access (post, browse newsfeeds, read articles) each social media outlet in a week? Please check the appropriate box corresponding to your estimated use. *(Checkboxes next to each option will include: Daily, 5-6 days/week, 3-4 days/week, 1-2 days/week, <1 day/week, Don’t use/have account)*

a. Twitter

b. Facebook

c. Instagram

d. Snapchat

e. LinkedIn

**Information about Fellowship Programs**

1. When researching fellowship programs, which resources did you use to obtain information? (check all that apply)

a. Social media

b. Fellowship website

c. Doximity

d. Co-residents

e. Current/Past Fellows

f. Attending mentors

g. Other

Please provide explanation of your selection of “other” in the previous question (optional) _________

2. When researching fellowship programs, which factors are the most important to find information on? Please rank from #1-9, with #1 being the most important

a. Application process

b. Curriculum (i.e. didactics, clinical rotations/rotation sites)

c. Faculty

d. Research opportunities

e. Sideline coverage opportunities

f. Fellowship alumni

g. Fellowship extras (i.e. paying for equipment, books, conferences)

h. Biographies of current fellows

i. Lifestyle of fellows (i.e. hobbies, out of hospital activities, info about city/area)

3. Do you feel that the information about fellowship programs available on program websites is adequate?

a. Yes

b. No

4. If you used social media to obtain information, what information were you hoping to find on the fellowship social media accounts? Check all that apply. *(skip if you did not use social media)*

a. Application process

b. Curriculum (i.e. didactics, clinical rotations/rotation sites)

c. Faculty

d. Research opportunities

e. Sideline coverage opportunities

f. Fellowship alumni

g. Fellowship extras (i.e. paying for equipment, books, conferences)

h. Biographies of current fellows

i. Lifestyle of fellows (i.e. hobbies, out of hospital activities, info about city/area)

5. What types of social media posts interest you the most when looking at fellowship programs on social media? Please rank, with 1 being the most interesting: *(skip if you did not use social media)*

a. Fellow life (out of office/hospital activities)

b. Fellow/faculty/alumni accomplishments (awards, recognitions)

c. Fellow educational opportunities (didactics, rotations, coverage)

d. Research (published articles, grants)

e. Other

Please provide explanation of your selection of “other” in the previous question (optional) _________

**Applicant Interaction with Fellowship Program Social Media Accounts**

1. Have you “followed” or “friended” any PCSM fellowship program social media accounts?

a. Yes

b. No

2. What is your reasoning for NOT “following” or “friending” PCSM fellowship accounts on social media? Check all that apply.

a. Unwanted attention to own personal account

b. No useful information

c. Unaware of social media presence of fellowship programs

d. No definite reason

e. I do not use social media

3. During fellowship application/interview season, did you ever delete/alter your social media profile (i.e. change profile name or privacy settings) out of fear of being searched by prospective residency or fellowship programs?

a. Yes

b. No

c. I do not use social media

4. Did a program’s social media presence have any influence on your perception of the program or intended ranking?

a. Yes

b. No

c. I do not use social media

5. Was this influence or impact on ranking negative or positive?

a. Negative

b. Positive

c. Both

d. I do not use social media

6. Please give further explanation of whether a program’s social media presence has any influence on your perception program or intended ranking? (optional) _________

|  | Study Population (%) | AMSSM Population (%) |
| --- | --- | --- |
| Gender  Male  Female | 65.5  27.5 | 71  29 |
| Race/ethnicity  White  Asian  Hispanic  Black | 65.5  15.5  10.3  6.9 | 68.6  12.9  3.9  5.1 |
| Primary Specialty  FM  EM  IM  Ped  PMR | 41.4  13.8  12.1  10.3  19 | 72  4  5.5  10.3  9.8 |

***Figure 5*** *Comparison of survey demographics to demographics of American Medical Society of Sports Medicine (Figures per the AMSSM 2022-2023 Annual Report)*
